# Privacy Aware Incentivization for Participatory Sensing

**DOI:** 10.3390/s19184049

**Published:** 2019-09-19

**Authors:** Martin Connolly, Ivana Dusparic, Georgios Iosifidis, Mélanie Bouroche

**Affiliations:** 1 Department of Information Systems, Cork Institute of Technology, Cork T12 P928, Ireland; 2 School of Computer Science and Statistics, Trinity College Dublin, Dublin 2, Irelandgeorge.iosifidis@tcd.ie (G.I.)

**Keywords:** participatory sensing, identity privacy, privacy preserving, incentive mechanism, incentivization, incentive compatibility, data truthfulness

## Abstract

Participatory sensing is a process whereby mobile device users (or participants) collect environmental data on behalf of a service provider who can then build a service based upon these data. To attract submissions of such data, the service provider will often need to incentivize potential participants by offering a reward. However, for the privacy conscious, the attractiveness of such rewards may be offset by the fact that the receipt of a reward requires users to either divulge their real identity or provide a traceable pseudonym. An incentivization mechanism must therefore facilitate data submission and rewarding in a way that does not violate participant privacy. This paper presents Privacy-Aware Incentivization (PAI), a decentralized peer-to-peer exchange platform that enables the following: (i) Anonymous, unlinkable and protected data submission; (ii) Adaptive, tunable and incentive-compatible reward computation; (iii) Anonymous and untraceable reward allocation and spending. PAI makes rewards allocated to a participant untraceable and unlinkable and incorporates an adaptive and tunable incentivization mechanism which ensures that real-time rewards reflect current environmental conditions and the importance of the data being sought. The allocation of rewards to data submissions only if they are truthful (i.e., incentive compatibility) is also facilitated in a privacy-preserving manner. The approach is evaluated using proofs and experiments.

## 1. Introduction

Participatory sensing is a form of crowdsourcing that enables service providers to capture environmental data from mobile device users. Participatory sensing has wide applicability in areas such as smart cities [1], air pollution monitoring [2] and health [3]. 

Privacy is a key consideration in the success of a participatory sensing system. Participants may share readings that reveal confidential data pertaining to, for example, participant location or behavior. Some potential participants may be deterred by the possibility of their privacy being compromised by this.

Cryptography and anonymization can be used to provide participant privacy, but this may be at the expense of the quality of the service provider’s dataset. Specifically, if the service provider has no access to participant identity, it will not be able to allocate rewards to attract and incentivize users, which may prevent it from achieving the critical mass of data it needs to provide its service. In addition, if the credibility of the data submitter cannot be assessed, the evaluation of the validity of submitted data becomes more difficult. 

At the same time, the key to the success of a participatory sensing campaign is to attract a critical mass of relevant data which meets the service provider’s quality requirements. This in turn requires the service provider to attract a sufficient number of participants. While participants may be willing to make data submissions, they will, in general, expect some form of tangible (monetary or non-monetary) reward in return; for example, to compensate them for costs such as battery consumption. 

Privacy preservation should not prevent the service provider from allocating the rewards it needs to offer to attract and incentivize users. The inherent conflict to be addressed when designing a privacy-preserving incentivization scheme is thus identified as being, on the one hand, the need to link participant submissions in order to reward them, and on the other, the need to break this link to ensure privacy preservation [4]. 

This paper presents Privacy-Aware Incentivization (PAI), a platform that seeks to address the privacy requirements of participants in a way that does not hinder the service provider’s ability to incentivize participants and allocate rewards to a data submission only if it is truthful (known as incentive compatibility). To this end, PAI incorporates incentivization and incentive compatibility schemes that reward participants and evaluate the truthfulness of submitted data, respectively, without requiring access to their personal details.

As the rewards offered by the service provider must be set at a level that matches current environmental conditions and current participation rates, the incentivization scheme used by PAI to motivate participation must facilitate the real-time adaption of these rewards. Such a scheme must also seek to optimize the consumption of the service provider’s budget and must give the service provider the flexibility to tune the importance and utility of the data being sought. Therefore, the incentivization scheme used by PAI must facilitate adaptive and tunable reward allocation (R1). 

To ensure identity privacy for participants, it is not only necessary to hide their real identity but also prevent the pseudonymous monitoring of their activities. This is achieved by ensuring that the service provider or a third party is unable to conduct an inference attack to gain further private information about participants such as their habitual behavior, location and trajectory. 

The first point of potential privacy vulnerability occurs when participants are making data submissions. The service provider should not be able to identify participants from the data submissions they make and should not be able to link multiple data submissions made by the same participant. In addition, data submissions must be protected for service providers to ensure that other participants, peers or service providers cannot access the data they have paid for. Thus, PAI must provide anonymous, unlinkable and protected data submission (R2).

Another key point of potential privacy violation is when a participant receives a reward. To prevent an inference attack, it is necessary to ensure that the service provider cannot identify a participant, cannot trace participant activity and behavior and cannot infer further information about that participant from the allocation of a reward. Therefore, PAI must provide untraceable and unlinkable reward allocation (R3). Finally, PAI must ensure that the service provider or a third party cannot conduct an inference attack when a reward is being spent and must therefore provide untraceable and unlinkable reward spending (R4).

In addition to meeting the requirements for privacy preservation and reward allocation, PAI must ensure that data submissions received by the service provider can still be evaluated to determine whether they are truthful. This must be achieved without impinging upon the participant’s privacy. For this reason, PAI must facilitate privacy-aware incentive compatibility (R5).

The requirement for adaptive and tunable reward allocation (R1) is met through the adoption of the Adaptive Reward Allocation (ARA) approach [5], a Lyapunov optimization-based model that adapts rewards when sudden changes occur in what are frequently fast-moving participatory sensing environments. In particular, the response rate to offers already made by the service provider and the data utility are used to adapt the level of reward.

PAI meets the requirements for anonymous, unlinkable and protected data submission (R2), untraceable and unlinkable reward allocation (R3) and untraceable and unlinkable reward spending (R4) through the adoption and extension of Identity Privacy Preserving Incentivization (IPPI) [6], a decentralized platform that issues untraceable and unlinkable rewards in exchange for anonymous and unlinkable data submissions to create a privacy-aware adaptive incentive-compatible incentivization scheme that is robust to inference attacks from semi-honest service providers and other potential attackers. To fully address the requirement for anonymous, unlinkable and protected data submission, the platform is refined to ensure that only the service provider has access to the data submissions that are intended for it.

The requirement for incentive compatibility (R5) is achieved through the development of a Data Truthfulness Estimation (DTE) algorithm. This algorithm uses the Maximum Likelihood Estimation (MLE) statistical method to estimate the probability of the data submission being valid (i.e., truthful).

**Figure 1:**
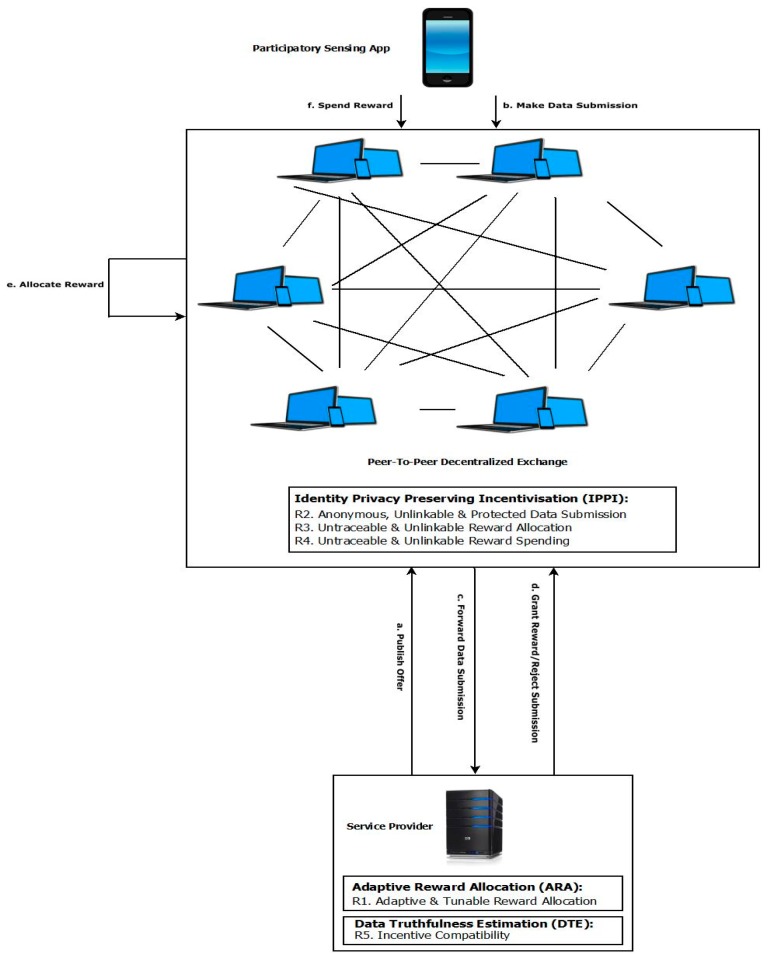
Privacy-Aware Incentivization (PAI) platform.

Figure 1 presents the PAI platform and the core components (IPPI, ARA and DTE) that are used to meet the requirements for privacy preservation, reward allocation and incentive compatibility, respectively. 

The evaluation of PAI builds upon proofs and experiments conducted for IPPI and ARA. The effectiveness of the DTE algorithm is evaluated by proof, while the performance evaluation of the cryptographic primitives used for privacy preservation are extended for both fixed and mobile devices, with a comparison with more recent work in the state of the art also being presented. The computational complexity of PAI is also evaluated in this paper.

- PAI is a decentralized platform that achieves the following:
  ○ Providing a data submission mechanism that ensures that data are submitted anonymously and cannot be linked to any data submissions previously made by the same participant.
  ○ Meeting the privacy requirements of the service provider by ensuring that the submitted data can only be accessed by this party.
  ○ Allocating rewards to participants without their having to cede their identity privacy. Specifically, rewards allocated to participants cannot be used to trace the activity of participants and cannot be used to link participants to their data submissions. In addition, rewards are also untraceable and unlinkable when participants spend them. This ensures robustness to inference attacks from service providers and other potential attackers.
  ○ Dynamically computing the rewards to offer to participants by adapting to participation rates and environmental conditions whilst seeking to optimize budget consumption. The ability to tune the scheme to balance data capture and budget consumption is also facilitated.
  ○ Incorporating a mechanism that assesses the truthfulness of data submissions made without violating participant privacy, thereby demonstrating that the approach facilitates incentive compatibility.
- An extensive evaluation of the approach is performed in terms of its privacy robustness, data truthfulness, performance and computational complexity.

It should be noted that this paper extends the work presented in [5] and [6] by the following:

(i) Providing an overall view of the challenges, requirements and the state of the art in privacy preservation, incentivization and incentive compatibility for participatory sensing;
(ii) Proposing an integrated architecture for a privacy-preserving and incentive-compatible incentivization scheme for participatory sensing;
(iii) Presenting the design and implementation of the Data Truthfulness Estimation algorithm;
(iv) Evaluating the overall platform against the most recent state of the art both in terms of performance and computational complexity.

The rest of this paper is organized as follows. Section 2 discusses related work and discusses how PAI progresses beyond the current state of the art. Section 3 outlines the system and threat models used for PAI. Section 4 presents the extended IPPI platform used to meet requirements R2–R4. The approaches taken for adaptive and tunable reward allocation (R1) and incentive compatibility (R5) are described in Section 5 and Section 6, respectively. Section 7 analyzes and validates the effectiveness of the approach taken by PAI in terms of the requirements identified, while Section 8 evaluates the platform’s performance. Section 9 concludes the paper.

## 2. Related Work

This section evaluates related work in the state of the art in terms of the requirements identified in the previous section.

### 2.1. Adaptive and Tunable Reward Allocation (R1)

While the state of the art includes many diverse approaches to incentivization and reward allocation, a significant proportion of them use an auction-based solution such as Vickrey–Clarke–Groves (VCG), sealed bid and reverse auctions ([7], [8] and [9] respectively). However, auctions entail a high level of overhead [10] as, in a participatory sensing environment, the service provider will typically need to gather all bids before deciding which participants to select. This in turn leaves participants vulnerable to privacy violations as, even if pseudonyms are used, the service provider can monitor participants’ bid activity. 

Some approaches use third-party components to manage reward allocation. However, these third-party components can themselves be points of privacy vulnerability. For example, one protocol [11] relies on the use of an additional component that manages the accounts of both participants and the incentive provider. This credit management component has particular potential for inference attacks as it performs credit transfers from the incentive provider’s account to that of the participant. 

Other approaches in the state of the art use statistical methods to implement their incentivization schemes and reward allocation. However, these approaches do not fully meet the requirement for adaptive and tunable reward allocation *(R1)*. For instance, the budget constrained simulated annealing technique (a probabilistic optimization method) that is used by EPPI [12] to minimize participant rewards does not consider the current participation rate and the dynamic environmental changes that may occur. Similarly, while the STOC-PISCES algorithm [13] considers data utility and participation rates when computing rewards, it does not take budget constraints into account. Finally, TLCI [14], which takes data quality and participation into account, does not facilitate tuning to prioritize efficient budget consumption over data capture and vice versa.

### 2.2. Anonymous, Unlinkable and Protected Data Submission (R2)

Balancing the needs of privacy preservation and incentivization is a challenge as, in some cases, the means of incentivization is itself a point of privacy leakage. For example, approaches in the state of the art that use pseudonyms, such as Stors [15], SPPEAR [16], PriCSS [17] and a certification center-based scheme [18], may prevent direct access to participants’ identities but can be used by the service provider to track activity and behavior. Some approaches do partially address the problem of pseudonym tracking. For example, one privacy-respecting auction [19] uses different pseudonyms in different auctions to ensure that bids made by the same participant in multiple auctions cannot be tracked. However, it is possible to track multiple bids made by a participant during a single auction.

Several privacy-preserving incentivization approaches proposed in the state of the art introduce third-party components to overcome the problems encountered through the use of pseudonyms. For example, one platform uses untraceable tokens and a Cloud provider [20], while ViewMap [21] uses the Tor browser to ensure participant anonymity (see https://www.torproject.org). However, as with those approaches in Section 2.1 that use third-party components for reward allocation, these components could themselves be a point of privacy violation through, for example, attacks, database leaks or seizure by governments [22]. This is acknowledged by one approach [23] which, when seeking to ensure bid privacy for its Vickrey–Clarke–Groves (VCG) auction-based scheme by grouping mobile participants’ bids, relies on a semi-honest third party. 

### 2.3. Untraceable and Unlinkable Reward Allocation (R3)

The medium for reward allocation is a crucial aspect to be addressed when designing a privacy-preserving incentivization scheme for participatory sensing. Reward allocation is typically addressed in the state of the art through the use of existing cryptocurrencies or the creation of reward tokens.

Cryptocurrencies are electronic forms of value exchange that have the potential to be used for online purchases, trading and transactions. Their name refers to cryptographic methods that are used to protect the transaction integrity as well as that of the currency itself. However, while the intention of cryptocurrencies such as Bitcoin [24] is to preserve the privacy of those engaging in transactions, the creators of Bitcoin point out that while the cryptocurrency provides anonymity, it is not pseudonymous; i.e., payees could be tracked through the address at which they receive Bitcoins. This address effectively functions as a pseudonym given that every transaction involving that address is stored in the BlockChain. For example, PaySense [25] uses the participant’s Bitcoin address as a pseudonym. While it may be possible to address this issue through the use of cryptocurrency ‘mixer’ services that enable users to swap Bitcoins with each other, this necessitates users placing their trust in what is often an anonymous third-party service. Another privacy-preserving incentivization platform [26], whose architecture necessitates the use of a third-party component, also uses Bitcoin to reward participants.

Alternatives to Bitcoin such as Monero (https://getmonero.org) and DASH (https://www.dash.org/) also have weaknesses that would prevent rewards from being untraceable. An empirical study [27] indicates that there are weaknesses in Monero in its use of fake coins, called mixins, to obscure transaction behavior but which, in fact, make transactions linkable under certain conditions as the mixins are sampled from a distribution that does not resemble real transaction inputs. In the case of DASH, an anonymous paper (See https://dashpay.atlassian.net/wiki/display/DOC/Dash+Security-Privacy+Paper), approved by the currency’s promoters, points out that DASH cannot be used securely and anonymously unless a third-party component such as the Tor anonymity network is used. In addition, the third party MasterNode mixing service provided by a third-party component increases the probability of tracing a payment, especially if there are few other users to swap coins with.

There are several approaches in the state of the art that use tokens, which are mappable to a tangible monetary or non-monetary item of value, to allocate rewards. However, this is sometimes at the cost of privacy preservation through the use of third parties ([18] and [11]) and pseudonyms [18]. One credit token system ([28], also [29] and [30]) does attempt to address the challenges of anonymous reward allocation by using a Blind Signature to break the link between the credit token and what is termed the pseudo-credit to ensure that the service provider cannot make any connection with the data submission for which the credit is earned. However, this approach does not address inference attacks as each credit token is directly linked to the participant’s ID. The authors themselves recognize this as they acknowledge the vulnerability of the approach to a credit-based inference attack. This is because the service provider may infer if participants have submitted data for a task from the number of credits that they have. 

EPPI ([12,31]) allocates rewards using token-based E-Cents—an exchangeable and untraceable unit bearer currency. What the authors refer to as a mix zone facilitates the anonymous exchange of E-Cents between participants in order to provide untraceability. However, it should be noted that the participant needs to use a pseudonym for this exchange service. Moreover, the service is itself a potential source of privacy compromise if it is illegitimately accessed. This is exacerbated by the fact that anonymity in this ‘mix zone’ is dependent upon the service achieving a sufficient number of participants and appears to rely upon manual intervention by participants. The privacy evaluation experiments carried out by the authors also indicate that the approach is, in certain circumstances, vulnerable to inference attacks. This is due to the use of what is termed an E-Cent pledge-based participating protocol which requires participants to submit E-Cents as a pledge that functions as a motivation for the participant to submit truthful data (as the participant will forfeit the E-Cents if they submit false data).

### 2.4. Untraceable and Unlinkable Reward Spending (R4)

A number of approaches in the state of the art provide untraceable and unlinkable reward allocation. For example, one credit token scheme [32] issues participants with a single token that accumulates rewards and is not linkable to a particular data submission. However, participants are required to reveal their identity when redeeming rewards, which means that their spending can be tracked. Similarly, while the credit tokens issued by another, third-party-free, scheme [33] cannot be linked to data submissions, participants are required to reveal their identity when depositing them. Indeed, the authors admit that there is a possibility of linkages with data submissions being made if a participant deposits multiple credit tokens simultaneously. While another approach [34] (an extension of [32]) does not appear to require participants to reveal their identity, the timing and pattern of reward token spending can nevertheless be monitored. 

### 2.5. Incentive Compatibility (R5)

Several approaches in the state of the art claim incentive compatibility by tracking participants’ reputations to evaluate the trustworthiness of data submitters. Typically, however, a reputation management system does not fully consider participants’ privacy requirements. Specifically, while there are several privacy-preserving trust and reputation management systems in the state of art that offer anonymity, they enable pseudonymous tracking of participant activity. For example, TATP [35] measures participants’ trust degree pseudonymously while PaySense [25] tracks participants through the Bitcoin account balances which are used to evaluate their reputation.

Other approaches use methods that evaluate data truthfulness but often do so at the expense of participant privacy. For example, participants’ reputation scores are fundamental to the truth discovery algorithm used by Theseus [36], which calculates the ground truth (i.e., the rate of data truthfulness) for each performed task. Similarly, the task assignment that is a core component of QEDE [37] requires direct access to participant activity. DECO [38], a false data detection and correction framework that applies a signal processing technique to reconstruct missing data and detect untruthful data, is specifically designed to evaluate data submissions from untrusted participants but is reliant on knowing the participant’s location to check data consistency. Similarly, the Social Incentive Mechanism [39] does not meet PAI’s privacy requirements as it leverages participants’ existing social relationships, while another approach [40] relies upon users to detect their malicious counterparts. SHIELD [41], which uses the Dempster–Shafer theory probability-based method as well as data mining techniques to detect untruthful data submissions, does not require direct access to participants’ personal details. However, it does require participants to register using a third-party identity and credential management component while the data verification framework itself is a third-party component—all points of privacy vulnerability. 

There are a number of approaches that do ensure data truthfulness without violating participant privacy. For example, one iterative approach [42] calibrates the sensing devices for the process of monitoring pollution sources by using the Expectation Maximization statistical method to create a model that evaluates the truthfulness of sensed sensor data. Specifically, the approach estimates whether the pollution source is present, the parameters that should be used for measuring the pollution level as well as the statistical noise caused by the sensing device without requiring access to a participant’s private information. As a result, this mechanism can be used to evaluate anonymous and unlinkable data submissions. 

### 2.6. Summary

To conclude, while there are many privacy-aware incentivization schemes in the state of the art, there is no approach that provides untraceable and unlinkable reward allocation (R3) and spending (R4) in return for an anonymous and unlinkable data submission (R2). For example, several schemes require private information from the participant in, for example, an auction bidding process, which makes data submissions linkable. Other approaches introduce third-party components that are themselves a potential point of privacy vulnerability as they hold participant details such as reward allocations. While other approaches do preserve direct identity privacy, they are nonetheless vulnerable to inference attacks through their use of pseudonyms that could be used to monitor participant behavior and activity and the issuing of traceable rewards, whether through the use of a cryptocurrency or reward tokens. 

The peer-to-peer distributed approach used by PAI, which avoids the need for third-party components, seeks to combat this potential for inference attacks by ensuring that each interaction between a participant and a service provider is stateless; i.e., each data submission made by a participant does not contain any means by which the service provider can link it to previous submissions made by the same participant. This is achieved by using a once-off untraceable ID for each data submission. As PAI does not require the use of third-party components or pseudonyms, the approach does not introduce any potential points of privacy vulnerability. Specifically, PAI uses one-off public/private key pairs for each data submission transaction to ensure that only a legitimate user can access the reward. The details of the data submission transaction are held by a peer who can neither identify the participant nor ascertain the content of the data submission.

The approaches taken by Li & Cao [28] and Dimitriou [34], both of which seek to provide anonymous reward allocation, are the approaches in the state of the art that are most similar in intent to PAI with respect to privacy preservation. While PAI seeks to progress beyond these approaches in providing untraceable and unlinkable reward allocation and spending, this should not come at an excessive performance cost. For this reason, these approaches will be used as the bases of comparison when evaluating PAI’s performance. 

There are a number of incentive compatibility approaches in the state of the art that ensure data trustworthiness but do so at the expense of privacy preservation. While the iterative incentive compatibility approach discussed in Section 2.5 [42] is intended only for pollution monitoring, the Expectation Maximization technique used has the potential to form the basis for a method that would meet PAI’s requirement for incentive compatibility without impinging upon its requirement for untraceable and unlinkable rewards in exchange for anonymous and unlinkable data submissions.

## 3. System and Threat Model

This section outlines the system (Section 3.1) and threat (Section 3.2) models used by PAI. 

### 3.1. System Model and Workflow

The participatory sensing model used by PAI is comprised of the participant and the service provider. The service provider will typically seek data that it will, for instance, publish or perform data analytics upon. Participatory sensing campaigns to capture this data are initiated by the service provider through the issuing of offers that specify what type of sensed data it is seeking (e.g., pollution levels in a particular urban location between 4 pm and 8 pm). Such an offer will also specify the reward that will be allocated to those participants who make data submissions that match the service provider’s criteria. 

Participants will typically own mobile devices such as smart phones, tablet computers, wearable devices or smart vehicles which have embedded sensors to capture data. The data captured by these smart devices can be scalar (e.g., temperature, air quality levels, GPS coordinates) or multimedia (e.g., photos, video). Once captured, a data submission is made to the service provider with a view to receiving a reward.

Once the service provider issues an offer, participants can then decide whether they want to respond to this offer. If the reward exceeds or equals the minimum reward a participant expects, that participant will then have to make a decision as to whether to submit data in response to this offer. Participants are assumed to be rational. It is thus expected that there will be a higher number of responses as the reward offered for a particular type of data is increased (this of course assumes that other factors—for example, privacy perceptions—are unchanged.). It is also assumed that costs are incurred by participants when they make data submissions. This could include, for instance, battery consumption or the consumption of the user’s mobile data allocation. These costs, as well as the sensing effort involved, necessitate participant incentivization.

In addition, the service provider’s budget is assumed to be finite and will be either of a monetary or tangible nature (for instance, Wi-Fi access). Rewards are only allocated to participants when they fully complete a task. 

The fundamental problem addressed by PAI’s incentivization scheme is one of time average cost minimization. This is because the service provider wishes to offer a reward that consumes the minimum amount of budget whilst still attracting an acceptable response rate from participants. This problem is modeled by assuming that the service provider operates in discrete time broken up into distinct slots t∈1, 2… The reward level is reviewed at the start of each of these timeslots. One or more offers seeking data submissions can be issued by the service provider in a particular timeslot, t. The service provider can elect to categorize offers using different granularity levels on the basis of, for instance, location accuracy. In addition to computing such rewards, PAI must also ensure that such rewards meet participants’ privacy requirements, both when the reward is allocated and when it is being spent.

The workflow of a typical participatory sensing system and the role of PAI’s requirements in each step is as follows:

- Firstly, the service provider makes a determination as to what type of data is being sought. To attract this data, it needs to also determine the level of reward that is most likely to attract its desired number of data submissions whilst at the same time not consuming an excessive amount of its budget. This challenge is addressed by the requirement for adaptive and tunable reward allocation (R1) which ensures that the reward is calculated on the basis of previous response rates for this type of data whilst also seeking to balance budget optimization and data capture. The service provider then publishes both the reward and the sought data on the OrderBook.
- While participants can view published offers on the OrderBook, they make be loath to respond to these offers if there is a possibility of their data submissions being used to track them or being linked to their previous data submission activity. This concern is addressed by the requirement for anonymous, unlinkable and untraceable data submission (R2). Under this requirement, participants that elect to respond to the reward will sense the data and generate a once-off One-Time Key which has no association with their identity. The public key component of the One-Time Key is used as an anonymous and untraceable ID for data submission while the data itself is encrypted using the service provider’s public key to ensure that only that party can view and access it. The response published on the OrderBook thus meets both the participant’s and the service provider’s privacy requirements.
- From the service provider’s perspective, the provision of privacy preservation for the participant makes it more difficult to evaluate the truthfulness of the data it receives given that its provenance cannot be determined. The requirement for incentive compatibility *(R5)* addresses this issue as, on publication of the response, the service provider will first decrypt the submitted data content and assess whether it meets the criteria outlined in its published offer. It then uses a data truthfulness method to determine whether the data submission is deemed to be truthful and thus merits a reward.
- If the service provider decides that the data submission should be rewarded, it publishes a confirmation on the OrderBook. PAI ensures that the service provider has no knowledge of who that party is and has no means of linking the public part of the One-Time Key to the participant through the fulfillment of the requirement for untraceable and unlinkable reward allocation (R3). The requirement for untraceable and unlinkable reward spending (R4) further addresses participant privacy as it ensures that the service provider cannot track the participant’s spending of the reward and/or link it to the data submission for which the reward was allocated. Specifically, the reward can only be spent by the holder of the private part of the One-Time Key; i.e., the participant who made the data submission.

### 3.2. Threat Model

The attack surface in a typical participatory sensing system is a large one with the sensing device, service provider infrastructure, third-party components used by the service provider and Internet communication all being potential points of attack. There are inherent threats, therefore, that have the potential to compromise participants’ privacy as well as the integrity of the data held by the service provider. Those parties who threaten the system (known as adversaries), the participants or the service provider can be either malicious or semi-honest in their intent. As the term implies, a malicious adversary intends to do harm to the system or a party within the system. On the other hand, a semi-honest adversary will be one of the parties within the system and follows the protocol specification exactly; however, it may try to learn more information than intended by examining data that it receives.

While there are a wide variety of attacks (both internal and external) that may target participatory sensing systems, PAI’s focus is on preventing the service provider from potentially using participants’ data submissions or allocated rewards to undertake inference attacks that will illegitimately grant access to further information about the participant; for example, the frequency of sensing activity in a particular location. The Semi-Honest Threat model is therefore the privacy model that must be addressed in order to meet the goals of PAI; specifically, to prevent inference attacks tracking the participant’s activity and behavior. In addition, under the PAI approach, the service provider is the only party who can access data submissions intended for it, thus ensuring that other participants cannot access this data. 

The Semi-Honest Threat model addresses privacy from the participant’s perspective. However, increased participant privacy also increases the potential for malicious participants to deceive the service provider and gain unearned rewards by submitting false or spurious data without penalty. This also affects participants who consume the service provider’s dataset as its overall quality is degraded. To combat this possibility and achieve incentive compatibility, PAI must also address the potential for False Data Injection Attacks by evaluating the truthfulness of submitted data. 

## 4. The PAI Platform

This section describes the PAI platform which refines IPPI [6] to take account of the service provider’s privacy requirements (As the privacy preserving requirements R2–R4 are fundamental to the platform, they are discussed before requirement R1 (adaptive and tunable reward allocation)) and incorporates mechanisms for reward computation and the enforcement of data truthfulness. The platform is used to fulfill privacy preserving requirements R2, R3 and R4. Section 4.1 discusses the provision of anonymous, unlinkable and protected data submission (R2) as well as describing the decentralized exchange platform that forms the basis for the approach. The means by which the allocation of participant rewards is made untraceable and unlinkable (R3) is then explored in Section 4.2. Lastly, Section 4.3 discusses untraceable and unlinkable reward spending (R4).

### 4.1. Anonymous, Unlinkable and Protected Data Submission (R2) 

Decentralized cryptocurrency exchanges such as CryptoNote are peer-to-peer networks that partition tasks pertaining to payment processing and recording among distributed computer systems. As such systems are decentralized, there is no central authority, middlemen or any other kind of third-party component [43]. This concept of a decentralized exchange has several characteristics that have the potential to support PAI in providing privacy preservation. Specifically, the distributed peer-to-peer application architecture enables the requirement for anonymous, unlinkable and protected data submissions to be fulfilled given that no private participant data are required as there is no direct communication between participants and the service provider. This prevents the service provider from tracing a participant through, for example, an IP address. Moreover, the approach does not use third party components (The potential occurrence of other external attacks such as Distributed Denial of Service (DDOS) attacks is also reduced as there is no central service. This means that all peer devices would have to be targeted.) that could potentially compromise participant privacy given that PAI uses distributed peers. Payments can be made or service access granted to these peers to motivate them to hold data submissions and reward information on behalf of the service provider.

**Figure 2:**
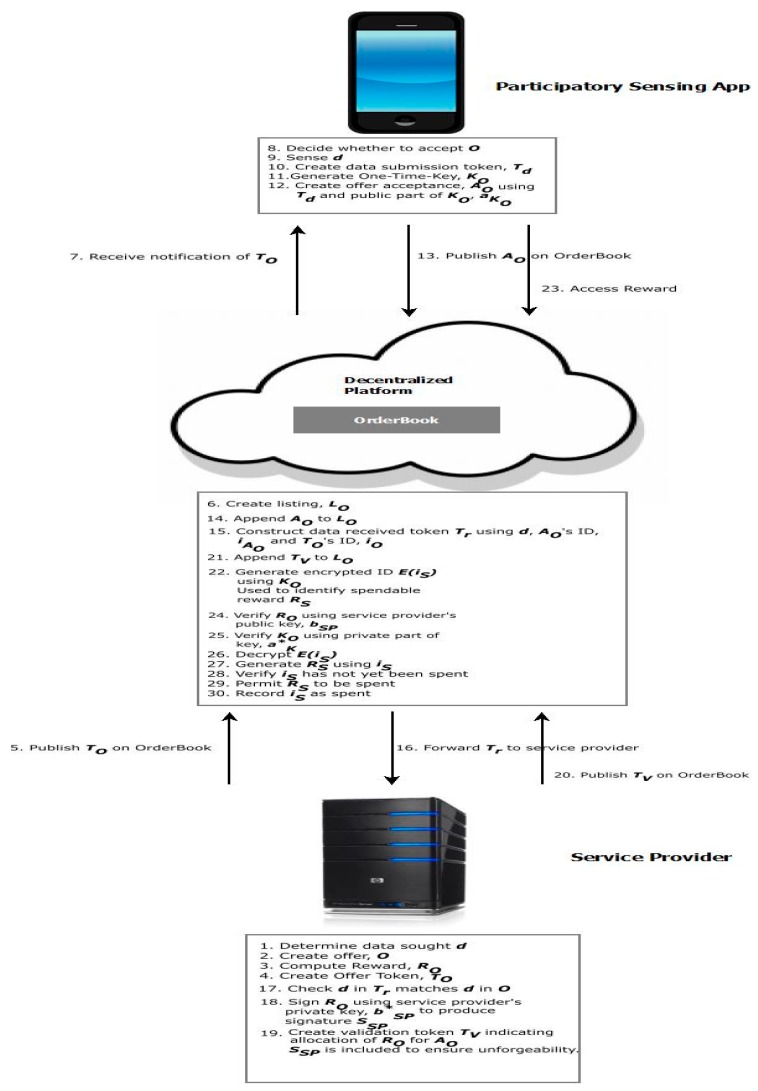
Privacy-Aware Incentivization platform.

While the fundamental distributed architecture used for decentralized cryptocurrency exchanges is unchanged, a number of modifications need to be made to the core concept of the OrderBook (which lists currency trades) to facilitate untraceable reward allocation for PAI. Specifically, the service provider publishes data submission requests and allocates rewards using the OrderBook, while, from the participant’s perspective, it facilitates anonymous data submissions and the receipt of untraceable rewards. Using the OrderBook, the service provider publishes offers that specify the kind of data that is being sought as well as the reward for a data submission of this kind. Participants are made aware of offers when they are published. They can then choose to respond to these by making a data submission as well as forwarding a token to which any reward will be allocated. The service provider allocates rewards until it has the number of responses it wants or until the expiration of the offer. Figure 2 presents an overview of how the PAI platform provides privacy preservation.

### 4.2. Untraceable and Unlinkable Reward Allocation (R3)

Cryptocurrency exchanges ensure unlinkability between multiple payments made to the same payee through the use of One-Time Keys [43] which themselves use the Diffie–Hellman Key Exchange Protocol [44]. This facilitates the establishment of a shared secret between two parties. This secret can then be used to exchange cryptography keys for use in symmetric encryption algorithms; for example, AES. 

PAI modifies this use of the Diffie–Hellman Key Exchange to create a One-Time Key that consists of a public and private key component. Both components are held by the participant with the public component being published on the OrderBook when the participant responds to an offer made by the service provider. This ensures that the service provider, who is analogous to the payer in a cryptocurrency exchange, cannot access the participant’s identity, be it real or pseudonymous. In this way, untraceable rewards are provided to participants and inference attacks are prevented.

Participants are informed of offers from the service provider when the latter publishes an offer token, $T_{O}$, on the OrderBook.

(1) $$T_{O}=\left\{\delta ,r_{O},i_{\mathrm{SP}},i_{O}\right\}$$

where δ represents the data that is being sought and specifies the type of data desired as well as other conditions; for example, data granularity, location, the number of data submissions sought and when the offer expires. $r_{O}$ denotes the reward offered by the service provider in exchange for a valid data submission, while the ID of the service provider and offer token are represented by $i_{\mathrm{SP}}$ and $i_{O}$, respectively. $T_{O}$ forms part of a listing, $L_{O}$, which is published on the OrderBook with participants’ responses being appended to $L_{O}$. 

On acceptance of $T_{O}$, a participant generates a One-Time Key, $K_{O}$, and uses the public part of $K_{O}$ to create an offer acceptance:

(2) $$A_{O}=\left\{d\;,\;a_{K_{O}},\;i_{O},i_{A_{O}}\right\}.$$

$A_{O}$ is comprised of the public part of $K_{O}$, $a_{K_{O}}$ (The symbols used correspond to those used in [44]), the participant’s data submission d, the ID of the corresponding offer token, $i_{O}$ and a unique ID, $i_{A_{O}}$. $i_{A_{O}}$ is assigned by the OrderBook when it receives $A_{O}$. It should be noted that no ID is assigned by the participant to $A_{O}$ as this would make the offer acceptance potentially traceable.

The PAI platform refines IPPI to take the service provider’s privacy requirements into account. Specifically, the service provider may not want a peer, or indeed another potential service provider, to view the data it is paying for. For this reason, the participant is required to encrypt d in its offer acceptance using the service provider’s public key $b_{\mathrm{SP}}$:

(3) $$A_{O}=\{\left\{d{\}}_{b_{\mathrm{SP}}},\;a_{K_{O}},\;i_{O},\;i_{A_{O}}\right\}.$$

$A_{O}$ is appended to the offer listing, $L_{O}$, on the OrderBook. It is then sent on to the service provider who, after decrypting ${\left\{d\right\}}_{b_{\mathrm{SP}}}$ using its private key $b_{\mathrm{SP}}^{*}$, determines whether the data submission merits a reward. To do this, the service provider determines whether d matches the specifications laid out in the offer token, $T_{O}$, and estimates whether the submitted data is truthful using the incentive compatibility approach outlined in Section 6. As the data submission contains no reference to the participant’s identity, the service provider cannot determine or otherwise infer who made the data submission. Once d is evaluated, a validation token is then created and published on the OrderBook by the service provider:

(4) $$T_{V}={\left\{i_{V},\;i_{A_{o}},v\right\}}_{b_{\mathrm{SP}}^{*}}$$

$T_{V}$ has a unique ID, $i_{V}$ and also consists of the offer acceptance ID $i_{A_{o}}$ as well a flag v that signals whether a reward should be allocated for this data submission. To confirm that $T_{V}$ indeed came from the service provider, that party uses its private key, $b_{\mathrm{SP}}^{*}$, to sign $T_{V}$. On receiving and verifying $T_{V}$, a unique spendable reward ID, $i_{S}$, is created by the OrderBook, with the public part of $K_{O}$, $a_{K_{O}}$ being used to encrypt a spendable reward token that can only be accessed by the participant holding the private part of $K_{O}$:

(5) $$r_{s}=\;{\left\{i_{S},i_{V}\;,\;r_{O}\right\}}_{a_{K_{O}}}$$

In addition to $i_{S},\;$ $r_{s}$ also includes the associated ID of the validation token, $i_{V}$, and the reward value, $r_{O}.$ Both $T_{V}$ and $r_{s}$ are appended to the offer listing $L_{O}$ on the OrderBook. 

### 4.3. Untraceable and Unlinkable Reward Spending (R4)

To spend a reward, a participant accesses $r_{s}$ from the offer listing, $L_{O}$. This is then decrypted using $a_{K_{O}}^{*}$, the private component of the One-Time Key $K_{O}$ that is held solely by this party. The decrypted reward is then sent to the OrderBook for spending. The OrderBook checks that the reward is indeed valid by verifying its provided ID, $i_{S}$, and also that it has not been spent before. In addition, the service provider’s public key, $b_{\mathrm{SP}}$, is used to verify the signature of the corresponding validation token, $T_{V}$. This is done to confirm that the service provider did in fact generate $T_{V}$. The reward can be spent once these verifications have taken place. The spending of the reward is logged in the OrderBook to prevent the problem of double spending. 

The allocation of a reward in return for a particular data submission can be viewed by all participants on the OrderBook. However, only the participant who holds the private part of the One-Time Key, $a_{K_{O}}^{*}$, corresponding to the public component, $a_{K_{O}}$, can decrypt the spendable reward, $r_{s}$. This decryption cannot be forged by other participants. It should also be noted that the OrderBook holds an identity certificate signed by a peer. This confirms that the service provider is the owner of the public key, $b_{\mathrm{SP}}$, that is used to verify rewards being spent and, in addition, ensures that the service provider cannot replace or otherwise change its signature with a view to tracing spendable rewards.

The algorithm for untraceable and unlinkable reward allocation is presented in Algorithm 1. This algorithm activates when a participant publishes an offer acceptance in response to an offer published by the service provider on the OrderBook. Algorithm 2 presents the algorithm used to ensure untraceable and unlinkable reward spending.

## 5. Adaptive & Tunable Reward Allocation (R1)

PAI has adopted the Adaptive Reward Allocation (ARA) approach [5] to enable the computing of rewards for offers made by the service provider. This section describes the mechanism. Section 5.1 discusses why Lyapunov optimization is chosen as the underlying method for reward computation. The budget consumption optimization problem is modeled in Section 5.2, while the design of the reward algorithm is described in Section 5.3. The incorporation of data utility is outlined in Section 5.4. The implementation of the algorithm is presented in Section 5.5. It should be noted that the reward computation mechanism does not require any access to participants’ private information. 

### 5.1. Lyapunov Optimization

The method that underpins PAI’s reward allocation mechanism, Lyapunov optimization, is used to facilitate the control of dynamic systems such as incentive and price computation in data communication networks. It has already been used in the state of the art for incentive design for participatory sensing [45] (but not, it should be noted, for reward computation). Its ability to respond to sudden and rapid changes that occur over time in the environment to which it is applied [46] make it particularly suitable for dynamic participant sensing environments as does its suitability for the minimizing of dynamic costs [47]. Such attributes are directly relevant to service providers given that these parties will typically seek to optimize their budget consumption in addition to capturing the most timely and relevant data. 

Any incentivization mechanism for a participatory sensing campaign will need to respond to sudden and rapid changes in an environment as this will often change the nature, quality, level of detail and accuracy of the data being sought. At the same time the consumption of the service provider’s budget must also be borne in mind. These factors can both be taken into account by a Lyapunov optimization model, thus making it particularly appropriate for PAI. This will also ensure adaptability as the reward is computed, taking both current participation rates and environmental conditions into account. It should also be noted that a service provider requires no access to future knowledge during a participatory sensing campaign; i.e., the response rate from participants. This is a further argument to use Lyapunov optimization, which makes no assumptions in this regard.

The most common use of Lyapunov optimization is for the allocation of resources in, for example, computer networking [48]. The use of the method must therefore be modified to meet the goals of the incentivization scheme required for PAI given that, when viewed as an economic market, participatory sensing has a number of distinct characteristics. Crucially, the dynamic and fast-changing nature of many participatory sensing environments means that the ‘product’ (i.e., the data being sought by the service provider) can potentially change suddenly. Moreover, the value of the data to the service provider at a particular point in time will change depending on that party’s needs. This is a key differentiating attribute in participatory sensing compared to other areas that require price optimization such as wind power or cloud infrastructure rental because, while demand changes in these markets, the actual product does not. The type of data sought may change over the course of a participatory sensing campaign and may only be relevant for a fixed period of time. In other words, its time-sensitive nature requires it to precisely match the information which is being sought by the service provider [49]. For these reasons, a market-based model is a suitable means of designing a participatory sensing incentivization scheme. 

**Algorithm 1**: Reward Allocation[Service Provider publishes an offer token $T_{O}$] //OrderBook operation. Append $T_{O}$ to offer listing, $L_{O}$. [On acceptance of $T_{O}$ by a participant] Capture d //Generate the public and private components of the One-Time Key. Generate $a_{K_{O}}$ and $a_{K_{O}}^{*}\;$
//Create offer acceptance, $A_{O}.$
$A_{O}=\{\left\{d{\}}_{b_{\mathrm{SP}}},\;a_{K_{O}},\;i_{O},\;i_{A_{O}}\right\}$ //Participant retains One-Time Key since the private key, $a_{K_{O}}^{*},$ is used to claim reward. //$\left[a_{K_{O}},a_{K_{O}}^{*}\right]$ represents the set of One-Time Keys held. $\left[a_{K_{O}},a_{K_{O}}^{*}\right]$ + = {$a_{K_{O}},a_{K_{O}}^{*}$} Publish $A_{O}$ on OrderBook //OrderBook Operation. $L_{O}+\;=\;A_{O}$//Append $A_{O}$ to $L_{O}$
Forward $A_{O}$ to Service Provider //Service Provider Operation. [On receipt of $A_{O}$] //Decrypt the data submission. Decrypt ${\left\{d\right\}}_{b_{\mathrm{SP}}}$ using $b_{\mathrm{SP}}^{*}$
v = Validate $A_{O}$
if v then    Log allocation of $R_{O}$//Make reward allocation.    $T_{V}={\left\{i_{V},\;i_{A_{o}},v\right\}}_{b_{\mathrm{SP}}^{*}}$
   $L_{O}+\;=$
$T_{V}$ //Append $T_{V}$ to $L_{O}$ and publishon OrderBook.    //OrderBook generates and publishes encrypted spendable reward.   $r_{s}$
$=\;{\left\{i_{S},\;i_{V},\;r_{O}\right\}}_{a_{K_{O}}}$
end if

**Algorithm 2**: Reward Spending[Participant wants to spend the reward] Decrypt $r_{s}$ using $a_{K_{O}}^{*}$
[OrderBook operation] //Verify that the associated validation token’s signature is a valid one from the service provider. Verify signature of $T_{V}$ (identified by $i_{V}$ entry in $r_{s}$) if verification passes then     Check that $i_{S}$ is not already recorded as spent     if $i_{S}$ is not recorded as spent then         Permit spending         Record $i_{S}$ as spent     end if end if

### 5.2. Budget Consumption Optimization Problem

This section formulates the Lyapunov optimization-based budget optimization problem. The method requires past response rates for different levels of reward and has no need for future information. The challenge to be met is to minimize the time average reward whilst still attracting the desired number of responses. In this way, budget consumption for the service provider is optimized. To facilitate ongoing reward computation, the participatory sensing campaign is modelled as a series of discrete timeslots with rewards being computed at the start of each timeslot. Thus, for any given timeslot *t*, the budget, *B* (Capitalization is used to correspond to the notation found in [50]), consumed by the service provider is

(6) B(t)=r(t)N(t)

The reward offered during a timeslot is denoted by r(t) while the number of responses received is represented by N(t). 

Like all Lyapunov optimization models, PAI’s reward computation scheme requires a control decision. In this case, this is the determination of an optimal reward level  r(t) for a timeslot *t*. As a result, the time average budget consumption for this policy is

(7) $$B_{\mathrm{AV}}≜\underset{t\to \infty }{\lim}\frac{1}{t}\overset{t-1}{\underset{t=0}{\sum }}E\left\{r\left(t\right)N\left(t\right)\right\}$$

Of course, while one of the key goals of PAI’s reward model is to compute a reward level that minimizes the time-average budget consumption, it must also ensure that the offered reward attracts a sufficient number of responses. Both factors must therefore be taken into account when designing the reward algorithm.

### 5.3. Designing the Reward Algorithm

Balancing budget consumption optimization and attaining the response rate desired by the service provider requires a trade-off between minimizing the number of offers that have been forfeited because the reward is too low and optimizing the consumption of the budget available to the service provider. To do this, a virtual queue, which does not exist in reality (it is a software implementation to facilitate the definition of the Lyapunov optimization-based model [50]), is defined to model the number of forfeited responses fir a particular timeslot, *t*, denoted by *Z*_forfeit_*(t)* (Z is used to denote a virtual queue. This notation corresponds to that used in [50]).

Determining the value for *Z*_forfeit_*(t)* in a given timeslot requires the service provider to set the number of desired responses, *N*_desired_. For a given timeslot, *t*, subtracting the actual number of responses received *N*_received_*(t)* from *N*_desired_*(t)* thus gives *Z*_forfeit_*(t):*

(8) $$Z_{\mathrm{forfeit}}\left(t\right)=\{\begin{array}{cc} 0 & N_{\mathrm{received}}\left(t\right)\geq N_{\mathrm{desired}}\left(t\right)\\ \min\{N_{\mathrm{desired}}\left(t\right),P\left(t\right)\}-N_{\mathrm{received}}\left(t\right) & N_{\mathrm{received}}\left(t\right)<N_{\mathrm{desired}}\left(t\right) \end{array}$$

An estimate of the number of participants in the system is represented by *P(t)*. *Z*_forfeit_*(t)* is the key determinant when computing the optimal reward for a timeslot *t*. For PAI’s reward computation mechanism, the Lyapunov Function for *t* is thus

(9) $$L\left(t\right)≜\frac{1}{2}Z_{\mathrm{forfeit}}{\left(t\right)}^{2}$$

This quadratic Lyapunov function gives a scalar (one-dimensional) measure for the total queue backlog for the participatory sensing system. Given the dynamic nature of the participatory sensing campaign, this Lyapunov function would be expected to change between timeslots. This is known as the one-slot conditional Lyapunov drift:

(10) Δ(t)≜L(t+1)−L(t)

Given the requirement for timely responses, the solution that best minimizes the Lyapunov drift and the associated queue backlog must be chosen for each timeslot; i.e., it must be greedily minimized. This ensures adaptive reward allocation and the balance required between minimizing the reward offered in exchange for a data submission (thus, optimizing budget consumption) whilst still obtaining a sufficient number of meaningful and timely responses. In queuing theory terms, the model seeks to achieve queue stability by continuously pushing the queue backlogs towards a lower congestion state. To optimize budget consumption, therefore, the Lyapunov drift expression must include the budget consumption term *B(t)*. This then leads to the following drift-plus-penalty expression:

(11) Δ(t)+V{B(t)}

The goal here is not only to minimize the queue backlog but also to minimize budget consumption. Thus, both should be minimized simultaneously. Under Lyapunov optimization, such a minimization objective is referred to as a penalty. The limit of the drift-plus-penalty expression, referred to as a bound, must be then minimized [51], which is the ultimate goal of Lyapunov optimization. In addition, a non-negative control parameter, *V*, is included so that the weighted budget consumption term is taken into account for the control decision. Thus, the trade-off between reducing the backlog of *Z*_forfeit_ and minimizing *B* is facilitated. In statistical terms, the upper bound for the drift-plus-penalty expression must be determined.

While a general case for a drift-plus-penalty bound [47] is presented, it must be extended for a participatory sensing environment. Assuming that $N_{\mathrm{received}}\left(t\right)$ is i.i.d (independent and identically distributed) over timeslots for any control algorithm seeking to minimize the allocated reward, *r(t)*, the following upper bound can included for the drift-plus-penalty expression used for the Lyapunov optimization-based problem being addressed here [51]:

(12) $$\begin{array}{c} \Delta \left(t\right)+V\left\{B\left(t\right)\right\}\;\leq B_{\mathrm{constant}}\left(t\right)+VE\left\{B\left(t\right)|Z\left(t\right)\right\}\\ +\;Z_{\mathrm{forfeit}}\left(t\right)E\left\{N_{\mathrm{received}}\left(t-1\right)-N_{\mathrm{desired}}\left(t\right)|Z_{\mathrm{forfeit}}\left(t\right)\right\} \end{array}$$

$B_{\mathrm{constant}}(t$) is a positive number used when carrying out the Lyapunov optimization computation:

(13) $$\;\;B_{\mathrm{constant}}\left(t\right)≜\frac{1}{2}{\left(N_{\mathrm{received}}\left(t-1\right)-N_{\mathrm{desired}}\left(t\right)\right)}^{2}$$

The goal here is similar to other Lyapunov optimization-based models [52] in that the objective is not to directly minimize the drift-plus-penalty expression but rather the right-hand side of the upper bound. For this reason, the queue backlog, $Z_{\mathrm{forfeit}}\left(t\right)$, is monitored in every timeslot *t*. This then enables the Lyapunov optimization approach [47] to be adapted so that the budget consumption *B(t)* can be chosen as the solution to the following problem:

(14) $$\;\min N\left(t\right)\left(Vr\left(t\right)+Z_{\mathrm{forfeit}}\left(t\right)\right)$$

The number of responses, *N(t)*, is determined by the reward offered, r*(t)*. Microeconomic supply curves are used to model this relationship; i.e., the response rate for a particular category of data submission at different reward levels. One of the rewards on the relevant curve that has been constructed for the current timeslot thus represents the solution for this minimization problem. In other words, there are a finite number of values for the reward to be allocated, *r(t)*, in a given timeslot *t*. The minimization problem is evaluated for all possible budget consumption levels with the reward that optimizes budget consumption then being selected. The service provider then publishes the offer token, $T_{O}$, with this reward. Once the responses are received, the relevant supply curve is updated to reflect the number of responses actually received. This process takes place for every timeslot during which an offer is published by the service provider.

While a Lyapunov optimization model only typically requires knowledge of the current state of the system, the algorithm used by PAI requires knowledge of the number of responses received in previous timeslots in computing *r(t)*. Higher rewards are thus offered when the backlog for *Z*_forfeit_ is large, while, conversely, lower rewards are offered in the case of a small backlog for *Z*_forfeit_.

Using standard Lyapunov optimization theory [52], a proof demonstrating the optimality of this minimization problem is presented:

**Theorem** **1.** **(Adapted from [47]).** $\underset{t\to \infty }{\lim}\underset{t\in Τ}{\sum }E\left(B^{†}\left(t\right)\right)\geq \;B^{*}-\frac{B_{\mathrm{constant}}}{\phi }$

*The budget consumption for a given timeslot,*t,*is denoted by*$B^{†}\left(t\right)$*, while*$\;B^{*}$refers to a budget consumption benchmark that assumes that the service provider can anticipate a defined number of possible scenarios; i.e., stochastic future information. The theorem shows that, as time tends towards infinity, budget consumption optimization converges to the minimum budget consumption, with a controllable error bound$O\left(\frac{1}{\phi }\right)$.

### 5.4. Incorporating Data Utility

In addition to being adaptive, the reward allocation scheme must also be tunable. Specifically, the scheme must tune the utility of the data being sought and balance this data capture with budget consumption. This can be achieved by modifying the value of *V* [50]. Assuming the time-average maximization problem’s objective value is $B_{\mathrm{av}}^{*}$ under an optimal policy, it is possible to present the following theorem [52]:

**Theorem** **2.** (**Adapted from [50]).** *For each timeslot, we assume the number of responses received in the previous timeslot*, $N_{received}\left(t-1\right)$*, and the number of desired responses in the current timeslot,*$\;N_{desired}\left(t\right)$*, are i.i.d. if there is an*γ >0*such that*:

(15) $$E\left\{N_{\mathrm{received}}\left(t-1\right)\right\}\leq \;E\left\{N_{\mathrm{desired}}\left(t\right)\right\}-\;\gamma$$

*It is then possible to present the following performance guarantees*:

(16) $$\underset{t\to \infty }{\lim}\frac{1}{t}\overset{t-1}{\underset{t=0}{\sum }}E\left\{p\left(t\right)N\left(t\right)\right\}<\;B_{\mathrm{av}}^{*}+\frac{B_{\mathrm{constant}}}{V}$$

(17) $$\bar{Z_{\mathrm{forfeit}}}≜\underset{t\to \infty }{\lim}\frac{1}{t}\overset{t-1}{\underset{t=0}{\sum }}E\left\{Z_{\mathrm{forfeit}}\left(t\right)\right\}\leq \;B_{\mathrm{constant}}+\frac{B_{\mathrm{constant}\;}+Vr_{max}P_{max}}{\epsilon }+\;\;\;\;N_{max}$$

In the case of PAI, the penalty that is incorporated to achieve queue stability in Lyapunov optimization is budget consumption, denoted by p(t). ϵ and $r_{\max}\;$ represent a constant > 0 and the maximum reward, respectively, while the number of responses received for $r_{\max}$ is denoted by $N_{\max}$.

Theorem 2 indicates that the larger the value for V, the closer budget consumption is to the optimal solution. However, increasing the value of V results in an increase in average queue backlog. As a result, budget consumption and the size of Z(t) require a trade-off. The service provider can tune this trade-off as it has the option to reflect the significance of the data being sought in a particular timeslot, t, by prioritizing data capture over budget consumption.

The tunability of the reward computation is facilitated by enabling the service provider to scale the importance of data it is seeking for a given offer. This is achieved by assigning a utility weighting value, *U*, for the data being sought. The value of the utility weighting maps directly to the significance of the data to ensure that the service provider can prioritize the capture of dynamic changes in the participatory sensing environment. Given that V increases as the data utility weighting, *U,* increases in order to prioritize data submission capture over budget consumption, the value of *U* maps to that of V as follows:

(18) $$U\propto \;\frac{1}{V}$$

In addition to tuning *U* to capture dynamic changes in the participatory sensing environment, it should also be noted that the predictive model that is used to construct the supply curve could also be used to tune utility by, for example, weighting the most recent data received if data are being sought in response to the most recent submissions that have been made. This would not require any modification of the reward computation algorithm. 

### 5.5. Algorithm for Adaptive and Tunable Reward Allocation

Algorithm 3 presents the algorithm for reward computation. Regression analysis is used to create the supply curves predicting the number of responses different rewards will generate. Table 1 presents the additional notations used in this algorithm. 

## 6. Incentive Compatibility (R5)

Economic theory states that when resources are being allocated among a group, individuals may find it in their interest to distort the information they provide so that they can acquire more of the resources than they should be entitled to [53]. These distortions in turn may lead to a suboptimal situation for the group as a whole as resources are inappropriately allocated. For participatory sensing, such a situation would occur if the service provider allocates rewards to participants who make data submissions that are not truthful. In this context, a truthful data submission is defined as one which accurately reflects the environmental measurement(s) being sought. An ‘untruthful’ data submission might not necessarily reflect dishonesty on the data submitter’s part (for example, the submission could contain inaccurate readings due to a hardware problem in the participant’s device) but nonetheless would not merit a reward from the service provider. The service provider suffers under this situation as its budget is wasted and the quality of its dataset is diminished. Other honest participants suffer as the budget for legitimate data submissions is effectively reduced so they could be deprived of rewards they would otherwise receive. The potential for participants to behave dishonestly is addressed by the economic concept of incentive compatibility, which addresses the design of an economic system whereby participants can achieve the best outcomes for themselves by acting truthfully [54]. Incentive compatibility is thus used for PAI to design a mechanism that statistically estimates whether a data submission is truthful or not.

The requirement for incentive compatibility (R5) is achieved for PAI by estimating the truthfulness of the data submitted to the service provider. A data submission can contain one or more categories of measurement values. As the majority of participatory sensing data are scalar numeric readings, it is this category of data that is addressed by the proposed incentive compatibility method. 

**Algorithm 3**: Computing the RewardCreate Supply Curve for $\left\{\left[N_{\mathrm{actual}}\right],\;r\right\}$ from Historical Dataset //Create Supply Curve. $M_{\mathrm{predict}}$ = Linear Regression Model for Supply Curve $\left\{\left[N_{\mathrm{actual}}\right],\;r\right\}$//Construct Linear Regression model. $\;\left[r\right]\;=\;0..r_{max}$ //Predict the responses for different reward levels. $\left[N_{\mathrm{predict}},r\right]$ = $predict\left(M_{\mathrm{predict}},\;\left[r\right]\right])$
foreach r //Construct the each reward’s queuing state variables.     $Z_{\mathrm{forfeit}}\left(t\right)=\;N_{\mathrm{predict}}\left(t\right)\;-\;N_{\mathrm{desired}}\left(t\right)$
    $\left\{\left[r,\;N_{\mathrm{predict}}\left(t\right),Z_{\mathrm{forfeit}}\left(t\right)\right)]\;\}+\;\left[r,\;N_{\mathrm{predict}}\left(t\right),\;Z_{\mathrm{forfeit}}\left(t\right)\right)\right]$
end foreach $B_{\mathrm{constant}}\left(t\right)=\;0.5\;*\;{\left(N_{\mathrm{received}}\left(t-1\right)\;-\;N_{\mathrm{desired}}\left(t\right)\right)}^{2}$//Compute Lyapunov Optimization constant. V = [U,V] //Map data utility weighting U to V foreach [$r,\;N_{\mathrm{predict}}\left(t\right),\;Z_{\mathrm{forfeit}}\left(t\right))]$ //Use Lyapunov Optimization to evaluate each reward.   $B\left(t\right)\;=\;r\;*\;N_{\mathrm{predict}}\left(t\right)/$/Compute the budget used by this reward.        if B(t) > $B_{{\mathrm{proportion}}_{\max}}$ then //Check that budget consumption does not exceed set maximum.        break    end if    $L\;=\;½\;*\;Z_{\mathrm{forfeit}}{\left(t\right)}^{2}$//Carry out the Lyapunov Optimization computation.    Δ(t) = $L-\;L_{last}\left(r\right)$//Compute one-slot conditional Lyapunov drift.    $DPP_{\mathrm{LHS}}$ = Δ(t) + (V * B) //Evaluate drift plus penalty expression.    $DPP_{\mathrm{RHS}}=\;B_{\mathrm{constant}}\left(t\right)\;+\;\left(V\;*\;B\right)$
$+\;Z\left(t\right)\left(N_{\mathrm{received}}\left(t-1\right)\;-N_{\mathrm{desired}}\left(t\right)\right))$
   if $\;DPP_{\mathrm{LHS}}\;>=\;DPP_{\mathrm{RHS}}$ then       continue    end if    $OPT_{\mathrm{current}}=\;N_{\mathrm{predict}}\;*\;\left(\left(V\;*\;r\right)\;+\;Z\left(t\right)\right)$//Evaluate the current optimization computation.    if $OPT_{\mathrm{current}}>\;OPT_{\mathrm{solution}}$ then       $OPT_{\mathrm{solution}}=\;OPT_{\mathrm{current}}$
      $r_{\mathrm{optimal}}=r$
   end if end foreach

There are a number of statistical methods that can be used to evaluate data truthfulness for scalar data. Section 6.1 discusses the choice of approach for estimating data truthfulness, while Section 6.2 describes the design and implementation of this approach.

### 6.1. Choosing an Approach to Estimate Data Truthfulness

As noted in Section 2.5, statistical techniques could be used to estimate data truthfulness without impinging upon the privacy requirements of the participant. In statistical terms, data truthfulness can be considered as a case of incomplete data; i.e., the truthfulness of the data submission cannot be known with 100% certainty. There are a number of statistical methods that can be used to make estimations for incomplete probabilistic models. However, the Gradient Descent algorithm (its loss function, used to compute the difference between, for example, a neural network output and ground truth, can be used for the purposes of evaluating data truthfulness) is reported to be slow [55] and would hinder both the scalability and performance of PAI while the Newton–Raphson method is dependent on the accuracy of an initial “guess”. The Method of Moments, another possible approach, has been found to be less precise than the Maximum Likelihood Estimation (MLE) method [56]. For this reason, MLE is used to quantify the correctness of data submission measurements.

Economic theory acknowledges that full incentive compatibility cannot be guaranteed for all categories of exchanges between parties [57]. This is the case in finite economies [58] such as a participatory sensing market in which there is a finite number of participants willing to make data submissions. In this case of limiting incentive compatibility, there is the potential for a party to gain from misrepresentation. This is the case here, as the MLE method computes a range that does not provide a definitive evaluation of data truthfulness but rather is used to estimate whether the data submission is truthful or not. 

### 6.2. Designing and Implementing an Approach to Estimate Data Truthfulness

Data truthfulness is assessed for each category of measurement value, c, received in the participant’s data submission, d. The algorithm exits if it considers any measurement value for a category, $m_{c}$, to be untruthful. If this occurs, the validation status, v, is assigned a value of  false and the validation token, $T_{V}$, published on the OrderBook denotes that the data submission is invalid. This, of course, then means that the participant will not receive a reward.

The initial step ensures that the value of the measurement for c, $m_{c}$, is within the minimum and maximum threshold limits, $m_{c_{\min}}$ and $m_{c_{\max}}$, set by the service provider for c. If it is not, v is assigned a value of  false. The next step in this process is to read the relevant data, [$d_{c}]$, that are held by the service provider for c. Depending on the measurement category, this could be the entire dataset, a subset of the dataset from a recent time period as determined by the service provider (for example, readings in the last hour), the last n number of readings where n is a number determined by the service provider, or the last n number of readings at, for instance, a particular time and/or location, The height, h, of the probability density function (PDF) for [$d_{c}]$ is then computed to assess how close the data values are to each other and is then used to formulate the natural logarithm to be used for the Maximum Likelihood Estimation (MLE) method, known as the Log Likelihood Function (LLF). Once initial estimates are set for the mean and standard deviation of the data value set ($\mu_{e}$ and $\sigma_{e}$ respectively), the MLE method is applied using these initial estimates and the LLF. The application of MLE results in the computation of an estimate for the mean, μ, and the standard deviation, σ, for which the normal distribution best describes this set of data values.

To prevent outliers and other potentially interesting (and valid) data being miscategorized as non-truthful data submissions and thus being discarded, the service provider can configure a scaling factor, $f_{\sigma }$, that is applied to σ to create $\sigma_{\mathrm{scaled}}$. By adding and subtracting ${\sigma \;}_{\mathrm{scaled}}$ to and from μ respectively, the scaled limits, $l_{\min}$ and $l_{\max}$, are computed for $m_{c}$. $m_{c}$ is then evaluated and if it is not between $l_{\min}$ and $l_{\max}$, v is assigned a value of  false. This process is repeated for all measurement categories [c]. If each $m_{c}$ is considered to be truthful, d is considered to be truthful and v is assigned a value of true.

Algorithm 4 presents the Data Truthfulness Estimation (DTE) algorithm used by PAI to estimate data truthfulness. This algorithm is applied for every measurement value contained in a data submission made to the service provider. The dynamic participatory sensing environment means that the data will be ever changing and evolving. As the dataset is changing with each data submission, the MLE parameters are thus either recomputed every time a change in the dataset occurs or periodically, with the service provider setting the recomputation interval in the latter case. This ensures that the incentive compatibility approach not only provides a means of estimating data truthfulness but does so in a way that reflects changes that have been captured in the service provider’s dataset. The configurable scaling factor also ensures that potentially interesting patterns and outliers that are reflected in incoming data streams are not inadvertently disregarded by the service provider. This further ensures that the requirement for the dataset to be reflective of changes in the dynamic participatory sensing environment is met. It should also be noted that the estimation of data truthfulness does not require any disclosure of identity privacy by the participant as the data submitted to the service provider contains no reference to the participant who made the submission. Therefore, while the participant who makes a non-truthful data submission does not receive a reward, the requirement for identity privacy is not violated.

It should be noted that the underlying MLE method has a number of limitations. For instance, it is only suitable for scalar data and cannot be used to evaluate the truthfulness of multimedia data content. This is unsurprising given that many statistical methods are only appropriate for scalar data. The effectiveness of MLE has also been found to be limited in a number of situations, for example, when the percentage of censored data (i.e., when the value of a measurement is only partially known) is large and the sample size is small. Nevertheless, it can be concluded that the data truthfulness estimation approach outlined here does fulfill the requirement for incentive compatibility (R5) as it seeks to demonstrate how incentive compatibility can be facilitated without impinging upon identity privacy.

**Algorithm 4**: Estimating Data Truthfulness[Service Provider receives a data submission d] //Evaluate data truthfulness for each category of measurement. foreach c in d
 //Check if the measurement value for this category is within the limits set by the service provider.  if $m_{c}$ < $m_{c_{\min}}$ or $m_{c}$ > $m_{c_{\max}}$ then   v = false //If not within the limits, set the validation status to false and exit the algorithm.   return v
 end if  Read data $\left[d_{c}\right]$ for c //Read the data for this category of measurement.  Get h from $\left[d_{c}\right]$’s PDF //Compute the height of the dataset’s PDF.  LLF = h−sum(log(h)) //Compute the Log Likelihood Function.  //Use initial estimates for the mean and standard deviation.  Use initial estimates $\mu_{e}$ and $\sigma_{e}$
 //Use MLE to estimate the two parameters (mean and standard deviation) for which the normal  //distribution best describes the data.  Compute μ and σ by applying MLE using LLF, $\mu_{e}$ and $\sigma_{e}$
 //Read the standard deviation factor for this measurement category, compute the scaled standard  //deviation setting and set the threshold limits.  Read $f_{\sigma }$
 $\sigma_{\mathrm{scaled}}$ = $\sigma \;*\;f_{\sigma }$
 $l_{\min}=\;\mu$ − $\sigma_{\mathrm{scaled}}$
 $l_{\max}=\;\mu$ + $\sigma_{\mathrm{scaled}}$
 //Check if the measurement value is within the threshold limits.  if $m_{c}$ < $l_{\min}$ or $m_{c}$ > $l_{\max}$ then   v = false//If not within the threshold limits, set the validation status to false and exit the algorithm.   return v
 end if end foreach v = true//If the loop has finished without exiting the algorithm, the data submission is a valid one. 

## 7. Analysis & Validation

This section presents proofs and experiments analyzing how PAI meets the requirements outlined in Section 1. The experiments validating the adaptiveness and tunability of the reward allocation scheme (R1) are presented in Section 7.1. Section 7.2 discusses how PAI fulfils the requirement for anonymous, unlinkable and protected data submission (R2), while proofs evaluating whether the requirements for unlinkable and untraceable reward allocation and spending (R3 and R4) are met are described in Section 7.3 and Section 7.4, respectively. The fulfillment of the requirement for incentive compatibility (R5) is analyzed in Section 7.5.

### 7.1. Adaptive and Tunable Reward Allocation (R1)

The requirement for adaptive and tunable reward allocation (R1) is evaluated in a simulated participatory sensing environment written using C++ and the Statistical R programming languages. The simulation is set up so that a series of offers is made by the service provider over its duration, with each offer seeking 100 responses. While service providers will seek a varied number of responses during participatory sensing campaigns, this figure is selected to clearly illustrate whether or not the level of reward being offered is adapting to the response. 

Offers are set to have a maximum reward level of 200 units. The duration of each simulation is 60 min with offers being published at 30-s intervals. It is assumed for the purposes of the simulation that one offer is made per timeslot. The level of reward is recomputed for each offer.

Two types of environment are configured for the simulation. What is termed the high response environment is, as its name implies, one in which there is a high initial number of responses from participants. The low response environment is one in which there is an initially low response rate from participants. The initial response rate is configured to be between 70% and 200% and 10% and 50% for high and low response environments, respectively, with a continuous uniform distribution being used to generate the participant response rate. A randomly generated increment is used by the simulation to vary the response rate and thus assess the adaptiveness of the reward to changes in the response rate. The incorporation of randomness is used to take other factors that may affect the response rate into account for the participatory sensing environment. A simple calculation is used for the response rate - the ratio of the number of submissions versus the number of responses sought.

**Figure 3:**
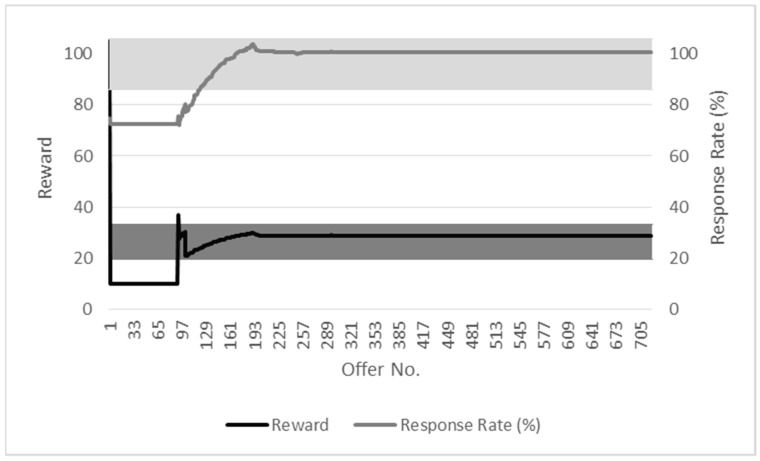
Reward adaptiveness: high response environment.

**Figure 4:**
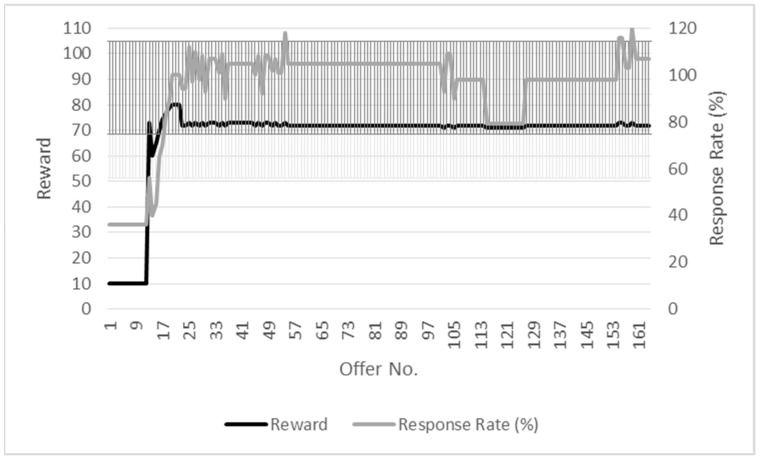
Reward adaptiveness: low response environment.

Figure 3 presents the adaptiveness of the reward allocation mechanism to the response rate in a high response environment. The average reward is 11.94 units for an average response rate of just over 101%. The reward is set to an initial figure of 105 units, but this rapidly decreases as the reward mechanism detects that the response rate is consistently high. This is further illustrated by the relatively low standard deviation figures of 2.78 and 4.08 for the reward and response, respectively. In contrast, Figure 4 shows the reward adaptiveness in a low response environment. The average reward of 66.13 units reflects the fact that the reward allocation mechanism has to take account of the initial low response rate. The successful adjustment of the reward to take such conditions into account is reflected in the average response rate of 92.98%. The standard deviations of 18.65 and 22.33, respectively, for the reward and response rate reflect the need for the reward allocation mechanism to increase the reward offered to improve the initial poor response rate.

### 7.2. Anonymous, Unlinkable and Protected Data Submission (R2)

R2 is demonstrated by showing that participants make data submissions to the service provider anonymously (Theorem 3), illustrating that the data submissions made by participants are unlinkable (Theorem 4) and showing that only the service provider can access these data submissions (Theorem 5).

**Theorem** **3.** *Data submissions made by participants to the service provider are anonymous*.

**Proof.**  For its participatory sensing campaigns, the service provider publishes offer tokens $\left[T_{O}\right]$ on PAI’s decentralized OrderBook to attract its desired data. Each offer token specifies the data being sought, δ, and the offered reward, $r_{O}$. The OrderBook publishes each offer token, $T_{O}$, as a listing, $L_{O}$, with participant acceptances,$\left[A_{O}\right]$, being appended to $L_{O}$. All participants and services providers can access the OrderBook. Participants can then decide whether to submit data, d, in exchange for the offered reward, $r_{O}$.

The service provider does not communicate with any of the participants when it is publishing the offer token, $T_{O}$. Likewise, there is no direct communication between a participant and the service provider when the offer acceptance, $A_{O}$ containing the data submission,  d, is published on the OrderBook given that $A_{O}$ is appended to $L_{O}$. The components making up, $A_{O}$ – $i_{O}$, the unique ID of the offer being responded to, the data submission, d, and the public component of the generated One-Time Key, $a_{K_{O}}$, cannot be connected to the participant’s identity but also cannot be connected to any anonymized ID or pseudonym that could be used to establish who the participant is. Furthermore, as the assignment of $i_{A_{o}}$ is done by the OrderBook, this ID cannot be used to trace the participant.

The service provider becomes aware of the data submission when it receives $A_{O}$. It can access and validate the data, d, within $A_{O}$ but cannot link the submission to the participant’s identity. It can therefore be concluded that data submissions made to the service provider are done so anonymously and do not reveal that participant’s identity Similarly, a peer listing the data submission cannot determine the participant’s identity from $A_{O}$. The participant can also take further steps to hide their device ID or, if necessary, their IP address through the use, for example, of a Virtual Private Network (VPN). □

**Theorem** **4.** *Participants make unlinkable data submissions to the service provider*.

**Proof.**  The public part of the One-Time Key, $a_{K_{O}}$, is used to identify the data submission made by a participant. In addition to containing no link to the participant’s identity, a different $a_{K_{O}}$ is generated for each data submission. As each data submission is an independent transaction with a different ID, the service provider has no means of linking data submissions made by the same participant. □

**Theorem** **5.** *Data submissions can only be accessed by the service provider*.

**Proof.**  When a participant publishes an offer acceptance, $A_{O}$, on the OrderBook, the data submitted is encrypted using the service provider’s public key, $b_{\mathrm{SP}}$. This encrypted data, ${\left\{d\right\}}_{b_{\mathrm{SP}}}$, cannot be accessed by any peer hosting the OrderBook or any other participant or service provider as it can only be accessed through decryption using the service provider’s private key, $b_{\mathrm{SP}}^{*}$. □

### 7.3. Untraceable and Unlinkable Reward Spending (R3)

R3 is demonstrated by showing that participants receive rewards that are untraceable (Theorem 6) and unlinkable (Theorem 7).

**Theorem** **6.** *Participants receive untraceable rewards*.

**Proof.**  The OrderBook publishes the public component of the One-Time Key, $a_{K_{O}}$, in the course of publishing the offer acceptance, $A_{O}$. As $a_{K_{O}}$ has no connection to the participant’s identity, it cannot be linked to the participant who created the associated One-Time Key, $K_{O}$, or be used to establish who the participant is.

To indicate whether $A_{O}$ merits a reward, a flag v for $A_{O}$ is published by the service provider on the OrderBook as part of a validation token, $T_{V}$. The intended recipient of the reward is indicated by $i_{A_{o}}$, the ID of the offer acceptance. As $i_{A_{o}}$ is generated by the OrderBook, it cannot be linked to the participant. On publication of $T_{V}$ by the OrderBook, it generates an encrypted spendable reward $r_{s}$ for $A_{O}$. $r_{s}$ can only be accessed and used by the participant who published $A_{O}$ as only this party holds the private part of the One-Time Key, $a_{K_{O}}^{*}$. It can therefore be concluded that participants can receive an anonymous reward in return for a valid data submission without their identity being divulged. □

**Theorem** **7.** *Participants receive unlinkable rewards*.

**Proof.**  A participant’s set of offer acceptances, $\left[A_{O}\right]$ cannot be linked as a One-Time Key, $K_{O}$, is only ever used once for a single offer acceptance, $A_{O}$. Furthermore, the ID of the spendable reward, $i_{S}$, cannot be linked to the participant or, indeed, to the public component of the One-Time Key, $a_{K_{O}}$, that was generated by that party given that this ID is randomly generated by the OrderBook. It should also be noted that as $r_{s}$ is only decrypted at the time of its spending, no connection can be made by the service provider between $r_{s}$ and its ID, $i_{S}$, to the participant entitled to this reward or to $a_{K_{O}}$. Thus, it can be concluded that data submitted by a particular participant cannot be linked using either $a_{K_{O}}$ or the $r_{s}$ encrypted using $a_{K_{O}}$.

A participant’s offer acceptances, $\left[A_{O}\right]$, public One-Time Key components, $\left[a_{K_{O}}\right]$, or spendable rewards, $\left[r_{s}\right]$, have no IDs that could be connected to that participant. As a result, the service provider has no means of making any inferences about the participant’s behavior and activity. □

### 7.4. Untraceable and Unlinkable Reward Spending (R4)

R4 is demonstrated by Theorem 8, which shows that participants’ reward spending is untraceable and unlinkable.

**Theorem** **8.** *Participants’ reward spending is untraceable and unlinkable*.

**Proof.**  The service provider plays no part in the publication of the offer acceptance, $A_{O}$, or in reward allocation. In particular, the service provider cannot use the assignment of this offered reward $r_{O}$ to track the activity of the participant who was allocated a reward. This is because the service provider cannot assign traceable IDs to $A_{O}$.

$A_{O}$ has no predetermined ID that can be used to track the participant as the OrderBook assigns a unique and random ID, $i_{A_{O}}$, when it receives $A_{O}$. Furthermore, the service provider cannot link participant activity from the publishing of that party’s offer acceptances, $\left[A_{O}\right]$, as the One-Time Key, $K_{O}$, is only ever used once and is never subsequently reused. In addition, there is no connection between the spendable reward, $r_{s}$, and $A_{O}$ as the former is only decrypted at the time of its spending. It can therefore be concluded that a participant’s set of spendable rewards, $\left[r_{s}\right]$, is untraceable and unlinkable. □

### 7.5. Incentive Compatibility (R5)

R5 is demonstrated by showing that data submissions that are considered to be non-truthful do not receive a reward (Theorem 9) and illustrating that the incentive compatibility method is privacy preserving (Theorem 10).

**Theorem** **9.** *Data submissions that are considered to be non-truthful do not receive a reward*.

**Proof.**  The service provider sets the minimum and maximum threshold limits, $m_{c_{\min}}$ and $m_{c_{\max}}$, and also configures a scaling factor, $f_{\sigma }$, that is used to compute the interval between the scaled limits, $l_{\min}$ and $l_{\max}$, that is deemed to contain valid values for a measurement category, $m_{c}$. Participants have no role in determining these parameters. Any measurement category in a sensed data submission d that fails either of these two tests results in d as a whole being considered untruthful and, consequently, not receiving a reward. □

**Theorem** **10.** *The incentive compatibility method is privacy preserving*.

**Proof.**  The service provider receives the sensed data, d, as part of the offer acceptance, $A_{O}$. Prior to approving the reward allocation, the service provider ensures that d matches the criteria of the offer represented by $i_{O}$ and can then evaluate the truthfulness of d using the data truthfulness enforcement method described in Section 6. While this ensures that the service provider does not allocate rewards for non-truthful data submissions, it does not violate identity privacy as the service provider has no access to the participant’s identity. At the same time, the fact that $T_{V}$ is published for every offer acceptance ID, $i_{A_{O}}$, ensures that participants can see that a non-truthful data submission has been rejected. □

## 8. Performance Evaluation

This section evaluates the performance of PAI, focusing on resource consumption in terms of time, energy and processing requirements. The most likely potential resource consumption bottleneck for any privacy preservation approach pertains to its use of cryptographic primitives. Thus, Section 8.1 evaluates the performance of those primitives used by PAI. To analyze the level of processing resources required by the algorithms as a whole, Section 8.2 assesses the computational complexity of PAI’s four algorithms. As outlined in Section 2.6, the approaches taken by Li & Cao [28] and Dimitriou [34] are used as the bases of comparison for evaluating PAI’s performance.

### 8.1. Cryptographic Primitives

As well as the C++ implementation of the algorithms for the simulated participatory sensing environment, PAI and the cryptographic primitives used for operations pertaining to the participant and OrderBook peers have been implemented for the Android mobile operating system using the Java programming language and the SpongyCastle API [59]. The digital signature is specified by the DSA algorithm with the SHA-1 algorithm being used for message digest. To address the possibility that a peer may choose to use a fixed rather than a mobile device (for example, a desktop computer or a PC server), the Java Programming language and the BouncyCastle API [60] are used to implement that party’s verification and decryption primitives for the Windows 10 operating system. As energy consumption does not tend to be a major concern for fixed devices, it is not measured for this latter implementation.

The implementation is deployed on a Samsung Galaxy S7 Edge Android smartphone (Android 7.0, 4GB RAM, Quadcore 2.3 and Quadcore 1.6 GHz CPU) and, in the case of a peer using a fixed device, on an 8GB Lenovo T450s ThinkPad laptop computer (8GB RAM, Intel Core i7-5600 2.6GHz CPU). The energy consumption and running time of the cryptographic primitives used for data submission and a typical peer are then measured. Table 2 presents the results of these experiments.

The cryptographic primitives used by the data submitter and the peer (both of which have to use resources when making data submissions) in PAI’s algorithms pertain to generation of the One-Time Key, the encryption of the data submission, the verification of the reward token by the peer when the user wants to spend the reward and the decryption of the spendable reward. To evaluate the running time of the cryptographic primitives used for PAI, the associated algorithm is executed over 100 times. The average time taken by the primitive is then computed. The time taken for the submitter when generating the One-Time Key and encrypting the data submission is 4.12 ms on average, while the verification process carried out by a peer (which takes place when the participant wants to consume the reward) and ID decryptions take under 1 ms. The comparison with participant resource consumption for the token-based approach proposed by Li and Cao [28] is favorable given that this method takes 12.5% longer on average. The overall time taken by the cryptographic primitives used by PAI is also substantially lower than that taken for participants in Dimitriou’s approach [34], whether the peer hosts the OrderBook on a laptop or a smartphone. The time taken by this approach is 257.5 and 3.8 times more than PAI when the peer hosts the OrderBook on a laptop and smartphone, respectively.

However, the 339 ms taken for the verification of the reward token by the peer is more expensive than Li and Cao’s approach when the peer hosts the OrderBook on a smartphone. With the data encryption and ID decryption taking 0.120 ms and 0.146 ms on average, respectively, the total running time for the cryptographic primitives of 343.266 ms is 57 times that of Li and Cao’s token-based approach [28]. The reward token verification is responsible for the majority of this cost, which is nevertheless well under half a second—a figure that is substantially lower than the 2 s commonly cited as the upper limit users expect for response time [61]. This verification process is a core part of the mechanism to decouple the reward allocation and reward spending process and thus ensures that, unlike Li and Cao’s approach, the data submitter cannot be the victim of inference attacks. Moreover, this only applies in the case of smartphone hosting of peer operations. If the service provider wants to encourage hosting on fixed nodes, it has the option to offer higher reward levels for those peers who do so. 

PAI’s smartphone power consumption for the data submitter is 71% lower than the approach taken by Li and Cao. It must be borne in mind that that the resource consumption totals for the approach proposed by Li and Cao (6.004 ms and 0.29 J, respectively) exclusively relate to the data submitter. When this is taken into account, PAI’s resource consumption of 4.12 ms and 0.025 J is significantly lower for the data submitter as most of the cost is borne by the peer. Energy consumption is also substantially lower than Dimitriou’s approach, at 3.8 and 504.48 times lower for a smartphone-hosted and laptop-hosted peer, respectively.

### 8.2. Computational Complexity

Computational complexity involves the study of the efficiency of algorithms based on the time and memory space required to solve a problem of a particular size [62]. Complexities are expressed using the Big O notation.

The majority of the computation for Algorithm 1 is of the order O(1); i.e., the costs of these operations are independent of the input. The generation of the digital signature by the service provider is dependent on the size of the key, $b_{\mathrm{SP}}^{*}$, used to sign $r_{O}$. Hence, using k to denote the size of $b_{\mathrm{SP}}^{*}$, the complexity of this operation is O(k). Similarly, the generation of $r_{s}$ (the encrypted spendable reward) and ${\left\{d\right\}}_{b_{\mathrm{SP}}}$ (the encryption of the data submitted) is dependent on the size of the message block, m, to be encrypted, so the complexity for these operations can be expressed as O(m). It should be noted that, in both cases, neither k nor m would be of a significant size as they entail digital signing using the service provider’s private key and the encryption of the spendable reward or data submission, respectively.

The runtime of the majority of the operations for generating the One-Time Key is O(1). The generation of the key pair used for the reward ID depends on the size of the keys. Assuming both keys are of the same size k, the computational complexity can be expressed as  O(2k). The most expensive part of the operation is the use of modular exponentiation, which entails the use of two digits of size n and an exponent of size k bits. A multiplication algorithm M is also used. The computational complexity of the modular exponentiation operation can be expressed as O(M(n)k) [63]. As this is the most expensive part of the operation, the Big O notation for Algorithm 1 can thus be expressed as O(M(n)k).

The most computationally expensive parts of Algorithm 2 pertain to the verification of $r_{O}$ and $K_{O}$ and the size of $b_{\mathrm{SP}}$ and $a_{K}^{*}$, respectively. Assuming the size of $b_{\mathrm{SP}}$ and $a_{K}^{*}$ are $k_{1}$ and $k_{2}$, the computational complexity can be expressed as $O\left(k_{1}\right)$ and $O\left(k_{2}\right)$, respectively. Likewise, the computational complexity when decrypting $r_{s}$ depends on the size of the message block m and so can be expressed as O(m). As this is the most expensive part of the operation, the Big O notation for Algorithm 2 can thus be expressed as O(m). The computational complexity of the core operations for Algorithm 3 depends on the range of rewards to be offered by the service provider; i.e., from zero to the maximum reward level, r_max._ Assuming the number of possible rewards the service provider could offer is |r|, the Big O notation for this aspect of Algorithm 3 can be expressed as O(|r|). The most expensive part of Algorithm 3 pertains to the linear regression method used to construct the supply curves for each category of measurement, c. Assuming that the number of elements for a particular c is $\left|n_{c}\right|$ and the number of terms for the linear regression equation is |l|, the computational complexity for the linear equation is $O\left({\left|l\right|}^{2}*\left(\left|n_{c}\right|+\left|l\right|\right)\right)$. This is because the underlying operation of linear regression entails matrix multiplication. Given that most offers will seek a finite number of measurement categories, |l| should be a comparatively low number.

Algorithm 4, which is used to estimate data truthfulness, is potentially a computationally expensive algorithm as its computational complexity is dependent on the number of measurement categories |c| and the number of items read from the dataset pertaining to this measurement category $\left|[d_{c}]\right|$. For a single category, therefore, the computational complexity can be expressed as O(|[$d_{c}]|$). Assuming the total number of items read from the dataset is |[d]|, the overall computational complexity of the algorithm can be expressed as O(|c||[d]|). The potential computational expense of Algorithm 4 is due to the need to read at least a subset of the dataset [$d_{c}]$ for each measurement category c to estimate the mean and standard deviation using the Maximum Likelihood Estimation (MLE) method. This expense can be offset by carrying out this computation periodically and by reducing the size of the subset to be read from the dataset.

The computational complexity of PAI compares favorably to that of Li and Cao ([28]) as the modular exponentiation scheme used for the RSA algorithm used by this approach has computationally expensive operations pertaining to the public key, private key and key generation, which can be expressed as $O\left(k^{2}\right)$, $O\left(k^{3}\right)$ and $O\left(k^{4}\right)$ respectively [64]. Thus, the Big O notation for Li and Cao’s approach can be expressed as $O\left(k^{4}\right)$, which is much larger than those for Algorithms 1 and 2 of PAI.

The computational complexity of EPPI [12] can be expressed in terms of its public key encryption and private key signature. Assuming the size of the E-Cent to be encrypted is s and that of the private key is k, the most expensive parts of the operation of this scheme can be expressed as O(k) and O(s), respectively. This compares favorably to both Li and Cao’s approach and PAI. However, depending on the asymmetric encryption scheme used for EPPI (for example, RSA), computational complexity can be up to $O\left(k^{4}\right)$, which is much larger than the worst case for PAI’s algorithms. Similarly, the computational complexity of PAI is favorable when compared with Dimitriou’s approach [34]. This approach relies upon zero-knowledge proofs which can have a computational complexity of O(|x|n) assuming a problem instance x and error probability $2^{-n}$ [65]. 

It should be noted that, while none of the approaches used for comparison have an algorithm that is potentially as computationally expensive as Algorithm 4, these approaches do not address the issue of incentive compatibility and data truthfulness evaluation.

## 9. Conclusions

This paper proposes PAI, a method of untraceable and unlinkable reward allocation and spending for participants in participatory sensing systems. PAI is a decentralized exchange platform that permits participants to make and receive rewards for data submissions without violation of their privacy. At the same time, the approach permits service providers to obtain the data they need to provide their service effectively. The adaptive incentivization scheme uses the Lyapunov optimization method to create an adaptive and tunable incentivization scheme with a view to providing rewards that capture data reflective of what is often a dynamic participatory sensing environment, while the Maximum Likelihood Estimation method is used to estimate the truthfulness of scalar data submissions. As PAI does not use pseudonyms or a trusted third party, it is robust to inference attacks from semi-honest service providers and other potential attackers and enables participants to be rewarded without violating identity privacy. 

This work addresses the key challenge of giving participants untraceable and unlinkable rewards in exchange for anonymous and unlinkable data submissions. Other challenges to be addressed include the evaluation of alternative methods to linear regression for predicting the response rate for a particular level of reward, the incorporation of a data truthfulness method for multimedia data submissions, the evaluation of system performance when more than one service provider participates in the system and/or multiple measurements are sought in a data submission, and the investigation of different means of motivating peer participation. By extending PAI to address the above challenges, it is possible that the approach could serve as the basis for a privacy-preserving marketplace for participatory sensing. 

## Figures and Tables

**Table 1:** Additional notations for Algorithm 3.

Notation	Meaning
DPP_LHS_	Drift-plus-penalty expression: left-hand side.
DPP_RHS_	Drift-plus-penalty expression: right-hand side.
L_last_(r)	Last Lyapunov function computed for a particular reward.
M_predict_	Linear Regression Prediction model.
OPT_current_	Current Lyapunov optimization computation.
OPT_solution_	Lyapunov optimization solution.
r_optimal_	The optimal reward value.
[r]	Possible reward values.
r(t)	The optimal level of reward to offer for a timeslot, *t*.
[r, N_predict_(t), Z_forfeit_(t)]	Reward, number of predicted responses and the associated queuing state variable for this reward.
{[r, N_predict_(t), Z_forfeit_(t)]}	The rewards, the number of predicted responses and their associated queuing state variables.
{[N_actual_], r}	Number of actual responses for the different levels of reward.
[N_predict_(t)], r]	Number of predicted responses for the different levels of reward.
[U,V]	The data utility weightings and the corresponding constant *V* (used to calculate the Lyapunov drift).
Δ(t)	One-slot conditional Lyapunov drift.

**Table 2:** Resource consumption of cryptographic primitives.

	Time (ms)	Power (J)
Submitter (Android Phone)		
- One-Time Key	4.000	0.023
- Data encryption	0.120	0.002
peer (laptop)		
- Verification	0.944	N/A
- Decryption	0.005	N/A
peer (smartphone)		
- Verification	339.000	3.290
- Decryption	0.146	0.002
peer (laptop)		
- Verification	0.944	N/A
- Decryption	0.005	N/A
peer (smartphone)		
- Verification	339.000	3.290
- Decryption	0.146	0.002
Total (laptop)	5.069	0.025
Total (smartphone)	343.266	3.317
Total (Li & Cao [28] Laptop)	5.704	N/A
Total (Li & Cao [28] smartphone)	6.004	0.290
Total (Dimitriou [34]	1305.500	12.612
